# Machine Learning Stratification of Periodontal and Cerebrovascular Disease Status Using Salivary and Subgingival Lipopolysaccharide Activity

**DOI:** 10.1111/jcmm.71337

**Published:** 2026-08-26

**Authors:** Anbo Dong, Zhenshan Xie, Muhammed Manzoor, Jaakko Leskelä, Jukka Putaala, Eija Könönen, Luigi Nibali, Pirkko Pussinen, Susanna Paju, Svetislav Zaric

**Affiliations:** ^1^ Centre for Host‐Microbiome Interactions, Faculty of Dentistry, Oral & Craniofacial Sciences King's College London London UK; ^2^ School of Biomedical Engineering & Imaging Science, Faculty of Life Sciences & Medicine King's College London London UK; ^3^ Department of Oral and Maxillofacial Diseases University of Helsinki Helsinki Finland; ^4^ School of Medicine, Institute of Dentistry University of Eastern Finland Kuopio Finland; ^5^ Neurology Helsinki University Hospital and University of Helsinki Helsinki Finland; ^6^ Institute of Dentistry University of Turku Turku Finland

**Keywords:** cerebrovascular disease, host‐microbiome interactions, lipopolysaccharide, machine learning, oral‐systemic axis, periodontitis

## Abstract

Oral dysbiosis may contribute to systemic inflammation, but taxonomic composition alone does not capture the host‐relevant inflammatory activity of microbial products. This study investigated whether salivary and subgingival lipopolysaccharide (LPS) activities could reflect host–microbiome interactions across periodontal and cerebrovascular disease states. Participants from the SECRETO Oral study were stratified by periodontal status and cryptogenic ischaemic stroke. LPS activity was quantified using a recombinant Factor C assay. Log‐transformed mean oral LPS activity and the subgingival‐to‐salivary LPS activity ratio were evaluated alongside demographic and behavioural variables in an exploratory machine‐learning framework. Integrated oral LPS activity increased across disease groups and was highest in participants with both periodontitis and stroke, while the LPS activity ratio differed across disease states, indicating altered niche distribution. Models combining demographic, behavioural and LPS‐derived features discriminated disease groups better than models using either feature set alone. LPS‐derived features therefore provided complementary, but not independently sufficient, discriminatory information. Oral LPS activity may represent a functional marker of microbial inflammatory burden across periodontal and cerebrovascular disease states. These exploratory findings require validation in independent cohorts with paired microbiome and endotoxin data.

## 1 Introduction

Cerebrovascular diseases, particularly ischaemic stroke, remain a leading cause of mortality and long‐term disability worldwide. Chronic inflammatory conditions contribute to vascular pathology through sustained immune activation, endothelial dysfunction and atherothrombotic processes [1]. Among these conditions, periodontitis has received considerable attention as a potential contributor to systemic inflammatory burden and cardiometabolic disorders and vascular outcomes, including stroke, partly through systemic exposure to microbial products and inflammatory mediators from inflamed periodontal tissues. Previous studies have linked periodontitis with cardiovascular outcomes, including atherosclerotic vascular disease and stroke, and support a role for periodontal inflammation in systemic vascular pathways [2, 3, 4, 5].

Oral dysbiotic microbial communities within subgingival biofilms induce persistent host immune responses, leading to tissue destruction and sustained inflammatory signalling [6]. Inflamed periodontal tissues may facilitate systemic exposure to microbial products and inflammatory mediators through microbial translocation, spillover of local inflammatory signals and endotoxemia [4]. These signals may activate circulating immune cells and promote systemic increases in pro‐inflammatory cytokines, including IL‐1β and IL‐6, thereby contributing to endothelial dysfunction, vascular inflammation and cerebrovascular vulnerability [7].

Recent investigations in stroke cohorts suggest that oral microbial composition may be associated with risk and severity of cerebrovascular disease [8]. However, taxonomic profiling identifies which microorganisms are present but does not directly capture the host‐exposed inflammatory bioactivity of microbial products. This distinction is important because microbial inflammatory potential is not determined by taxonomic composition alone; even closely related Gram‐negative taxa may differ in the quantity, structure, and immunostimulatory potency of their lipopolysaccharide. Functional measurement of endotoxin activity may therefore provide complementary information by capturing a host‐relevant microbial burden signal at the host–microbiome interface.

Lipopolysaccharide (LPS), a key microbial‐associated molecular pattern (MAMP) derived from Gram‐negative bacteria, plays a central role in host–microbiome interactions through activation of Toll‐like receptor 4 (TLR4) and downstream inflammatory pathways ([4, 9]). Beyond its canonical immunostimulatory role, endotoxin activity may reflect host exposure to microbial inflammatory burden, integrating signals across complex microbial communities. In periodontal disease, this functional readout complements taxonomic profiling by reflecting microbial inflammatory bioactivity rather than community composition alone [8, 10].

Subgingival LPS activity reflects site‐specific periodontal microbial burden, whereas salivary LPS activity represents a broader whole‐mouth signal [11]. Integrating these signals may therefore provide a more comprehensive representation of the oral host–microbiome interface.

Despite evidence that endotoxin activity reflects the microbial inflammatory burden, it remains unclear whether integrated oral endotoxin signals across distinct niches can capture host–microbiome interactions that link periodontal and cerebrovascular diseases. Therefore, this study aimed to evaluate whether combined salivary and subgingival endotoxin activity differed across combined periodontal and cerebrovascular disease states and whether LPS‐derived measures provided discriminatory information within a machine‐learning framework. We hypothesised that individuals with both periodontitis and stroke would exhibit higher integrated oral endotoxin activity than individuals without either condition, and that LPS‐derived measures would help discriminate between combined periodontal and cerebrovascular disease states.

## 2 Materials and Methods

### 2.1 Study Design and Population

Participants were derived from the SECRETO Oral study, a sub‐study of the international multicentre SECRETO study (Searching for Explanations for Cryptogenic Stroke in the Young; NCT01934725), which has been previously described [12]. SECRETO is a prospective case–control study of young adults aged 18–49 years with first‐ever imaging‐confirmed cryptogenic ischaemic stroke (CIS) and age‐ and sex‐matched stroke‐free controls. CIS was defined according to the A‐S‐C‐O classification system, and individuals with a definite identified stroke aetiology were excluded [12, 13].

For this analysis, participants were recruited from the Helsinki University Hospital and Turku University Hospital sites, with oral examinations performed between April 2014 and February 2020. Periodontal status was assessed using periodontal pocket measurements and panoramic radiographic evaluation of alveolar bone loss according to the 2017 World Workshop classification, as previously described [8, 12, 14]. Participants were then categorised according to cerebrovascular disease status and periodontal status for the four‐group machine‐learning analysis. All participants provided written informed consent, and ethical approval was obtained from the Ethics Committee of the Helsinki and Uusimaa Hospital District.

### 2.2 Data Preparation and Machine‐Learning Analysis

Participants were classified into four groups according to periodontal and cerebrovascular disease status. For machine‐learning analysis, to achieve balanced class representation, participants were randomly sampled within each group from the eligible cohort using a fixed random seed [15], resulting in equal group sizes (*n* = 34 per group) and a final analytical sample of 136 participants (Figure S1). This approach was adopted to minimise class imbalance and ensure stable model training. The sample size was constrained by the number of eligible participants within each group, and no formal sample size or power calculation was performed, as the study was designed as an exploratory machine‐learning analysis. Categorical variables were encoded using one‐hot encoding, and numerical variables were standardised.

Endotoxin activity was measured in salivary and subgingival samples using the EndoZyme II GO recombinant Factor C assay (bioMérieux, France) as previously described [11]. All samples were measured in duplicate, with laboratory personnel blinded to clinical diagnosis and group allocation. The resulting activity levels were used to construct LPS‐derived features for the machine‐learning framework. Primary models integrated demographic/behavioural variables and LPS‐derived variables, and additional feature‐set comparisons were performed to evaluate the discriminatory contribution of LPS‐derived variables alone and in combination with demographic/behavioural variables.

### 2.3 Microbiome Compositional Analysis

To further explore the microbial compositional context of subgingival endotoxin activity within each disease‐status group, available subgingival 16S rRNA gene sequencing data generated and processed as described previously were analysed [10]. Genus‐level centre log‐ratio (CLR)‐transformed abundances were correlated with log10‐transformed subgingival LPS activity within each group using Spearman correlation, with Benjamini–Hochberg false discovery rate correction applied within groups.

### 2.4 Model Evaluation and Interpretation

Machine‐learning models were used to evaluate whether oral endotoxin activity could stratify individuals across combined periodontal and cerebrovascular disease states. The input feature set integrated both demographic/behavioural variables (age, sex, BMI, smoking, alcohol use, hypertension and diabetes history) and endotoxin‐derived features. To represent the oral endotoxin burden, features were summarised as the log‐transformed mean endotoxin activity (LPS_log_mean) and subgingival‐to‐salivary endotoxin activity log‐ratio (LPS_log_ratio). LPS_log_mean was calculated as the average of log‐transformed salivary and subgingival LPS activity. This measure was intended to capture the combined endotoxin signal across the two oral niches, rather than relying on either salivary or subgingival LPS activity alone. A higher LPS_log_mean therefore reflects a higher overall oral endotoxin burden across groups. LPS_log_ratio was calculated as log‐transformed subgingival LPS activity minus log‐transformed salivary LPS activity, which is equivalent to the logarithm of the subgingival‐to‐salivary LPS activity ratio. This measure was used to indicate whether the endotoxin signal was relatively more represented in the subgingival or salivary compartment. By including these diverse parameters, the modelling framework accounted for potential confounding effects of established risk factors while evaluating the additive discriminatory value of microbial inflammatory markers.

Four supervised algorithms were evaluated: logistic regression with elastic‐net regularisation [16], linear support vector classifier (SVC), random forest and gradient boosting (XGBoost) [17]. An ensemble model combining predictions from the individual classifiers using weighted voting was also evaluated [18].

### 2.5 Statistical Analysis

Participant characteristics were compared across the four study groups using one‐way analysis of variance (ANOVA) for continuous variables and the chi‐squared or Fisher's exact test for categorical variables, as appropriate, with a two‐sided *p* value < 0.05 considered statistically significant. Model performance was assessed using stratified five‐fold cross‐validation and evaluated using accuracy, macro‐averaged F1 score and macro‐averaged area under the receiver operating characteristic curve (AUROC). Feature importance was estimated using permutation importance. Decision curve analysis (DCA) was performed to assess the potential clinical utility of the models by comparing their net benefit against two baseline strategies: ‘treat all’ (assuming all individuals require intervention) and ‘treat none’ (assuming no individuals require intervention) [19]. To assess the incremental discriminatory value of the endotoxin‐derived features, three feature sets were compared within the same modelling pipeline while holding all other settings constant: demographic and behavioural variables only, LPS‐derived variables only, and the combined feature set (Table S1). All analyses were performed using Python (version 3.14) with the scikit‐learn and XGBoost libraries.

## 3 Results

### 3.1 Study Population Characteristics

Participants were classified into four groups based on periodontal status (periodontitis vs. no periodontitis) and cerebrovascular status (stroke vs. no stroke) characteristics are summarised in Table 1. Significant differences were observed for endotoxin‐related variables, age, sex, smoking status, heavy alcohol use and hypertension (*p* < 0.05), whereas body mass index and diabetes history did not differ across groups.

**TABLE 1:** Participant characteristics of participants across the four study groups.

Variable	No perio/No stroke	No perio/stroke	Perio/No stroke	Perio/Stroke	*p*
Integrated oral LPS activity (log‐mean)	3.3 ± 0.2	3.5 ± 0.3	3.7 ± 0.2	3.8 ± 0.2	< 0.001
Subgingival‐to‐salivary LPS activity ratio (log‐ratio)	−0.3 ± 0.5	−0.7 ± 0.6	−0.8 ± 0.4	−0.8 ± 0.4	< 0.001
Age	38.1 ± 8.5	32.9 ± 8.4	43.2 ± 6.4	43.1 ± 4.7	< 0.001
Sex (Male/Female)	7/27 (20.6%/79.4%)	18/16 (52.9%/47.1%)	28/6 (82.4%/17.6%)	24/10 (70.6%/29.4%)	0.001
BMI	25.5 ± 4.0	26.6 ± 3.9	27.7 ± 4.9	27.9 ± 5.0	0.116
Current smoking (No/Yes)	31/3 (91.2%/8.8%)	16/18 (47.1%/52.9%)	25/8 (73.5%/23.5%)	17/17 (50.0%/50%)	0.001
Heavy alcohol use (No/Yes)	32/2 (94.1%/5.9%)	15/19 (44.1%/55.9%)	29/5 (85.3%/14.7%)	28/6 (82.4%/17.6%)	< 0.001
Hypertension (No/Yes)	30/4 (88.2%/11.8%)	24/10 (70.6%/29.4%)	24/10 (70.6%/29.4%)	13/21 (38.2%/61.8%)	< 0.001
Diabetes (%)	0%	0%	5.9%	2.9%	0.611

*Note:* Values are presented as mean ± standard deviation (SD) for continuous variables and *n* (%) for categorical variables. *p* values were calculated using one‐way ANOVA for continuous variables and either chi‐squared or Fisher's exact test for categorical variables, as appropriate. Perio denotes periodontitis, and Stroke denotes cryptogenic ischaemic stroke.

Both composite endotoxin measures showed a clear gradient across disease states. Integrated oral LPS activity (log‐mean) increased progressively across groups, reaching the highest levels in individuals with both periodontitis and stroke. In contrast, the subgingival‐to‐salivary LPS activity ratio (log‐ratio) decreased across disease groups, suggesting altered niche distribution of oral endotoxin activity rather than increased subgingival predominance. Although these group‐level differences were statistically significant, confidence intervals overlapped between adjacent groups, indicating that integrated LPS activity distinguished groups at the population level but did not fully separate individual participants.

### 3.2 Microbiome Compositional Analysis

In the exploratory microbiome compositional analysis, genus‐level CLR‐transformed subgingival microbial abundances were correlated with log10‐transformed subgingival endotoxin activity within each disease‐status group. Several group‐specific correlation patterns were observed after FDR correction (Figure S2 and Table S3). In the Periodontitis−/Stroke + group, subgingival LPS activity showed positive associations with *Selenomonas* and *Veillonellaceae* (*G‐1*), and negative associations with genera including *Haemophilus*, *Actinomyces* and *Rothia*. In the Periodontitis+/Stroke+ group, negative associations were observed for *Pseudopropionibacterium*, *Actinomyces*, *Kingella* and *Lautropia*.

### 3.3 Machine‐Learning Model Performance

**FIGURE 1:**
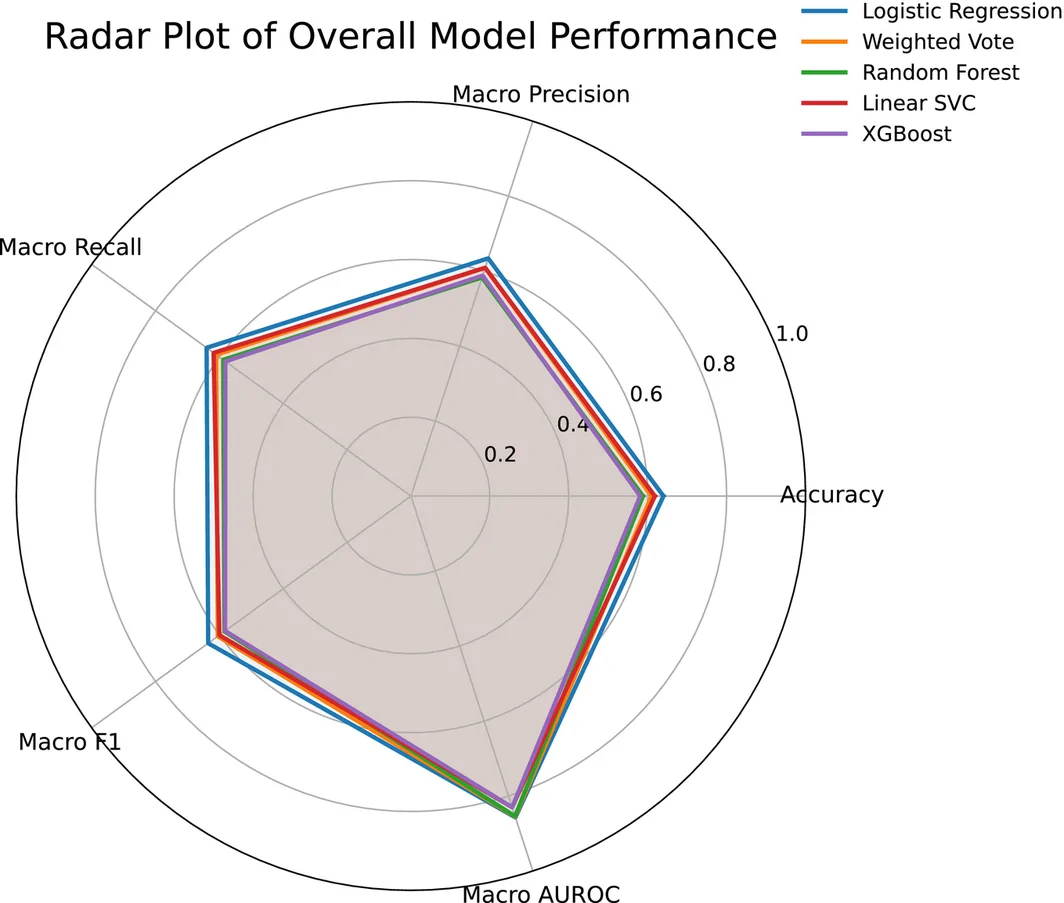
Comparison of classification performance across machine learning models. Radar plot comparing classification performance across machine learning models, including logistic regression, linear support vector classifier, random forest, gradient boosting (XGBoost) and a weighted voting ensemble. Metrics include accuracy, macro precision, macro recall, macro F1 score and macro AUROC.

Model performance is summarised in Figure 1. All models demonstrated comparable discriminative ability, with macro‐AUROC values ranging from 0.83 to 0.86. Performance metrics were derived from a stratified five‐fold cross‐validation. Logistic regression achieved the highest accuracy (0.64) and macro‐F1 score (0.635), while the ensemble WeightedVote model showed similar performance (accuracy 0.61; macro‐F1 0.605). Feature‐set comparison analyses showed that the combined demographic/behavioural and LPS‐derived feature set generally achieved the highest overall performance across models (Table S1). In the weighted voting model, the combined feature set achieved an accuracy of 0.610, macro‐F1 of 0.605, and macro‐AUROC of 0.855, compared with 0.522, 0.523 and 0.752 for demographic/behavioural variables alone, and 0.471, 0.435 and 0.748 for LPS‐derived variables alone. These findings suggest that LPS‐derived features provided incremental discriminatory information when combined with demographic and behavioural variables, although LPS‐derived variables alone showed modest classification performance.

### 3.4 Receiver Operating Characteristic Analysis

**FIGURE 2:**
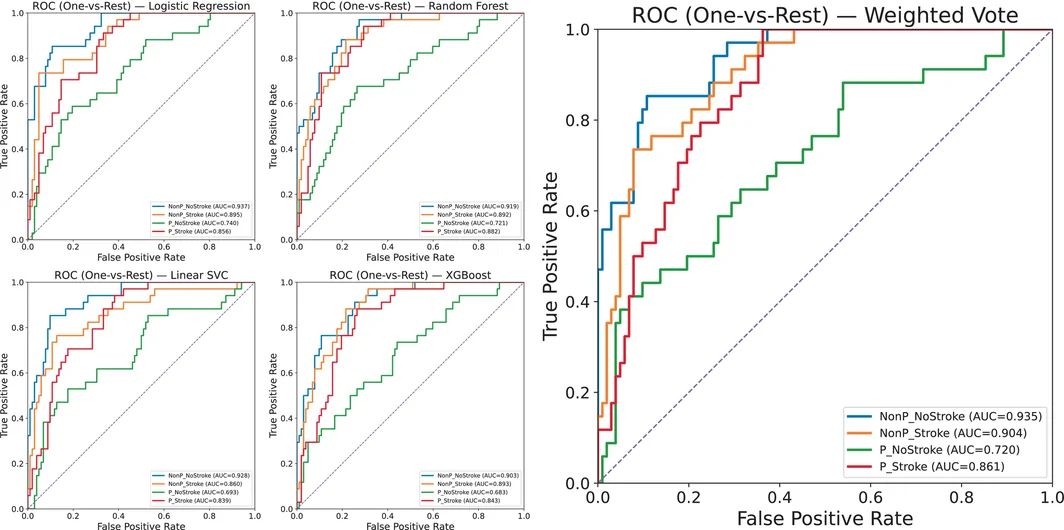
Receiver operating characteristic (ROC) curves for classification models. Class‐specific ROC curves are shown for each of the four disease groups and for each classification model, including the weighted voting ensemble. The corresponding area under the curve (AUROC) reflects discriminative ability for each group, with curves further from the diagonal reference line indicating better discrimination.

**FIGURE 3:**
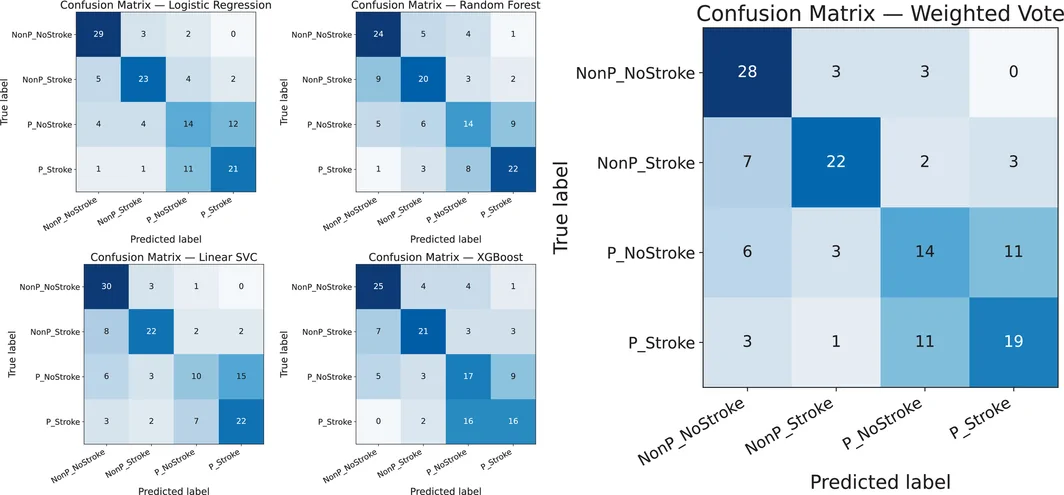
Confusion matrices illustrating the classification performance of the models. Each matrix shows the agreement between predicted and true group labels, with diagonal cells representing correct classifications and off‐diagonal cells representing misclassifications among the four disease groups. Periodontitis participants with and without stroke were most frequently misclassified as one another.

Receiver operating characteristic (ROC) curves for the evaluated machine‐learning models are shown in Figure 2. Logistic regression and the weighted voting ensemble demonstrated the strongest discrimination for individuals without periodontitis or stroke, as well as for periodontitis patients with stroke, with several class‐specific AUROC values exceeding 0.90. In contrast, classification performance for periodontitis patients without stroke was consistently lower across models, with AUROC values ranging from approximately 0.68–0.74. Confusion matrices are shown in Figure 3. Periodontitis participants with and without stroke were most frequently misclassified as one another, suggesting that oral LPS activity reflected periodontal inflammatory burden more strongly than stroke‐specific status.

### 3.5 Feature Importance Analysis

**FIGURE 4:**
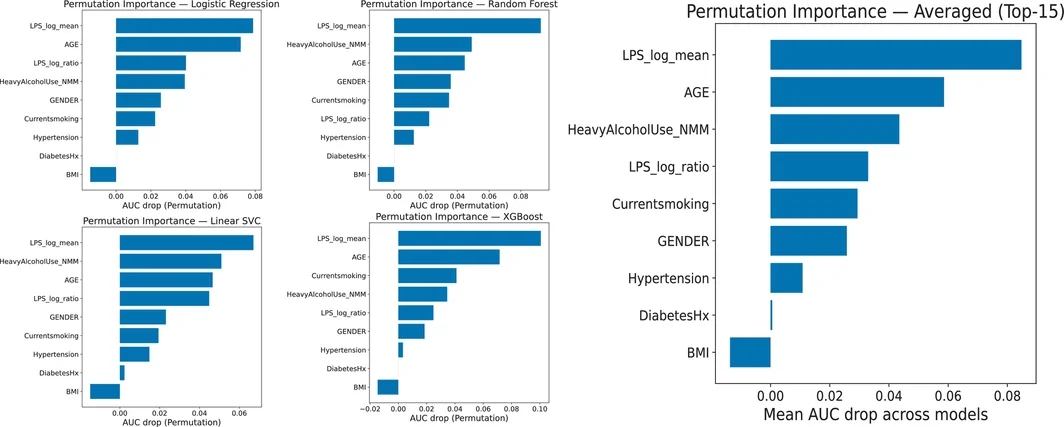
Permutation feature importance shows the relative contribution of variables across machine learning models. Importance is expressed as the mean reduction in macro‐AUROC when each feature was randomly permuted, with larger reductions indicating a greater contribution to classification. The log‐transformed mean endotoxin activity (LPS_log_mean) showed the largest reduction (approximately 0.08), followed by age (approximately 0.06), indicating modest effect sizes with no single dominant predictor.

Permutation feature importance analysis showed that endotoxin‐related features consistently ranked among the most influential predictors (Figure 4). In particular, log‐transformed mean endotoxin activity (LPS log‐mean) showed the highest importance across models. Age and heavy alcohol use also contributed to classification, whereas diabetes history and body mass index showed minimal importance. The magnitude of these effects was nonetheless modest: the largest reduction in macro‐AUROC for any single feature was approximately 0.08 (LPS_log_mean), followed by age, indicating that no individual feature dominated classification.

### 3.6 Decision Curve Analysis

**FIGURE 5:**
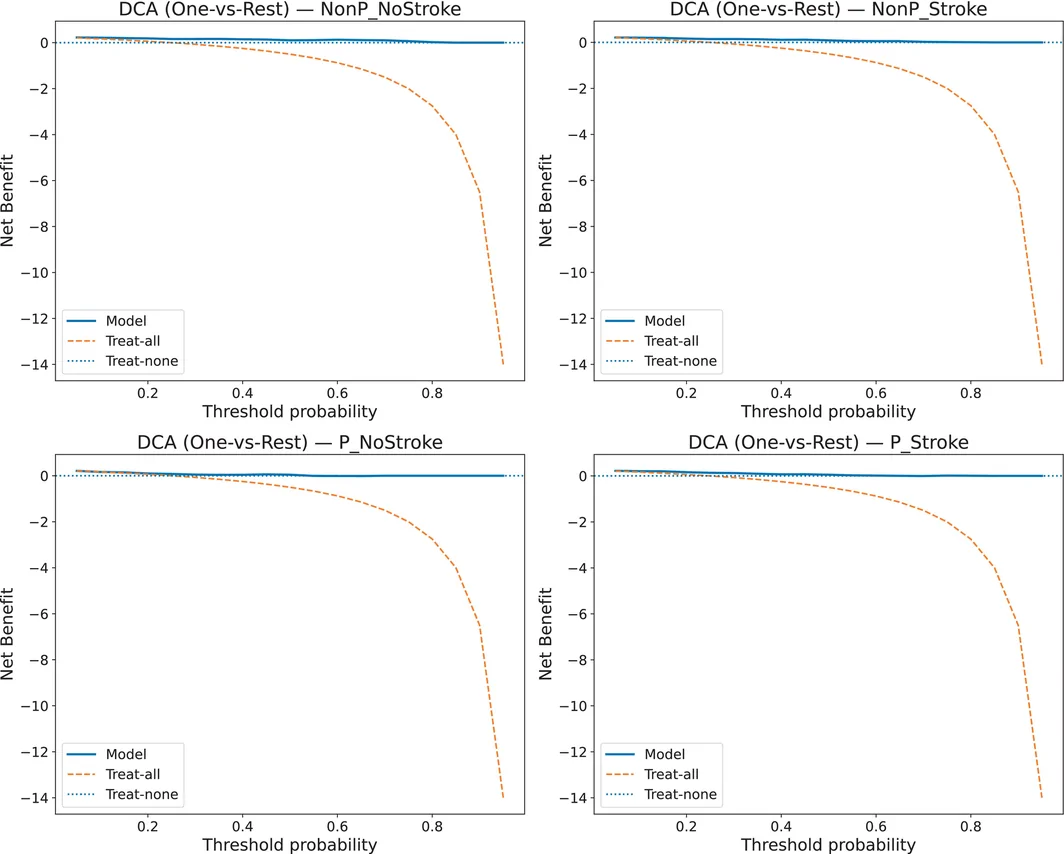
Decision curve analysis showing net clinical benefit of the weighted voting model across a range of threshold probabilities. Net benefit is plotted against threshold probability and compared with the treat‐all and treat‐none reference strategies. The model generally exceeded the treat‐all strategy, whereas its net benefit over the treat‐none strategy was limited across several thresholds; confidence intervals were not estimated.

Decision curve analysis compared the net benefit of the ensemble model with the treat‐all and treat‐none strategies across a range of threshold probabilities, which represent assuming that all or no individuals would receive intervention, respectively (Figure 5). The ensemble model generally exceeded the treat‐all strategy; however, its net benefit over the treat‐none strategy was limited across several thresholds. Confidence intervals were not estimated, and the raw net‐benefit values are provided in Table S2; these results do not establish clinical utility.

## 4 Discussion

This study investigated whether oral endotoxin activity measured in salivary and subgingival samples could stratify individuals according to combined periodontal and cerebrovascular disease status using a machine‐learning framework. Oral endotoxin activity differentiated the disease groups, and the log‐transformed mean endotoxin activity ranked among the most influential predictors, suggesting a functional interface between microbial dysbiosis and systemic inflammatory burden rather than a simple marker of local disease.

Lipopolysaccharide is a key microbial mediator linking periodontal inflammation with systemic immune responses relevant to cerebrovascular disease [4]. As a potent activator of innate immune signalling, endotoxins may contribute to low‐grade systemic inflammation associated with both periodontal and vascular pathology, thereby supporting a biologically plausible link between local oral dysbiosis and stroke‐related inflammatory processes [4, 8, 20].

This endotoxin signal is rooted in the underlying microbial community, as LPS derives predominantly from Gram‐negative bacteria. In the same cohort, the subgingival and salivary microbiota differ by disease status, with *Treponema denticola* and *Tannerella forsythia* enriched in stroke patients [8], and that endotoxin activity was associated with selected taxa in subgingival and salivary samples [10]. Consistent with this broader microbiome–endotoxin relationship, the exploratory microbiome compositional analysis in the present study showed that subgingival endotoxin activity was related to genus‐level community structure across disease‐status groups. These findings suggest that endotoxin activity may capture community‐level microbial inflammatory potential rather than the abundance of individual taxa alone. Importantly, taxonomic composition alone may not fully capture host‐relevant inflammatory activity, whereas endotoxin activity reflects the functional consequence of microbial communities [6]. Endotoxin activity measured across oral niches, therefore, captures complementary aspects of microbial exposure, with subgingival endotoxin activity representing local periodontal burden and salivary endotoxin activity reflecting broader whole‐mouth exposure [11]. Integrating these signals may provide a more comprehensive representation of oral microbial inflammatory burden.

Both periodontal disease and cerebrovascular disorders arise from multiple interacting factors, including microbial burden, host immune responses and cardiometabolic risk profiles [21]. Consistent with this, neither demographic variables nor LPS‐derived variables discriminated well in isolation, whereas combining them substantially improved performance, indicating that oral endotoxin activity and demographic factors carry complementary rather than redundant information. The comparable performance across algorithms further suggests a stable biological signal rather than model‐specific effects [22, 23]. The decision‐curve analysis, however, did not demonstrate a clear net benefit over the treat‐none strategy across thresholds [19].

From a biological perspective, oral endotoxin activity is an attractive, minimally invasive readout of microbial inflammatory burden that is relevant to both periodontal and systemic disease processes. These findings are consistent with the concept of oral endotoxemia as a potential link between local dysbiosis and systemic inflammatory processes, possibly mediated through the dissemination of microbial products from inflamed periodontal tissues. However, the present results should be interpreted as supporting further biomarker investigation rather than as establishing clinical utility, and they do not justify clinical decision‐making at this stage. Confirmation in larger, prospective cohorts, ideally larger cohorts with integrated paired microbiome and endotoxin data, would be required before any clinical application could be considered.

Several limitations should be considered when interpreting the findings of this study. First, the cross‐sectional design precludes causal inference regarding the association between oral endotoxin burden and cerebrovascular disease status. Second, although balanced group sampling was used to minimise class imbalance, the overall sample size was relatively modest, which may affect model stability and limits the interpretation of the machine‐learning findings. Third, the study population was derived from young adults with cryptogenic stroke within the SECRETO cohort, which may limit generalisability to older populations and other stroke subtypes. Fourth, although endotoxin measurements were performed in duplicate by laboratory personnel blinded to clinical status and group allocation, and demographic and behavioural covariates were included in the modelling framework, residual confounding remains possible because comprehensive data on other oral conditions, systemic comorbidities, socioeconomic status and health behaviours were not available. External validation in larger independent cohorts incorporating paired microbiome and endotoxin data is required to confirm the robustness and biological interpretation of these findings.

## 5 Conclusion

In conclusion, oral endotoxin activity derived from salivary and subgingival samples showed group‐level differences across combined periodontal and cerebrovascular disease states. In the exploratory machine‐learning analysis, LPS‐derived features provided modest complementary discriminatory information when combined with demographic and behavioural variables, but were not sufficient as stand‐alone classifiers. Decision curve analysis did not establish clinical utility. These findings should therefore be interpreted as exploratory and hypothesis‐generating, and further studies incorporating paired microbial‐composition data and external validation are required to clarify the biological and discriminatory relevance of oral endotoxin activity.

## Author Contributions

**Zhenshan Xie:** methodology, conceptualization, data curation, validation, formal analysis, writing – review and editing. **Eija Könönen:** data curation, investigation, writing – review and editing, project administration, funding acquisition, resources. **Jukka Putaala:** data curation, investigation, writing – review and editing. **Susanna Paju:** data curation, investigation, supervision, funding acquisition, project administration, resources, writing – review and editing. **Luigi Nibali:** writing – review and editing, resources, funding acquisition, supervision. **Pirkko Pussinen:** data curation, conceptualization, methodology, supervision, writing – review and editing, project administration, resources. **Jaakko Leskelä:** data curation, investigation, writing – review and editing. **Muhammed Manzoor:** data curation, investigation, writing – review and editing, formal analysis. **Anbo Dong:** conceptualization, methodology, software, data curation, validation, formal analysis, resources, writing – original draft, visualization. **Svetislav Zaric:** supervision, writing – review and editing, investigation, conceptualization, methodology, data curation, validation, visualization, project administration, resources, funding acquisition.

## Funding

This study was supported by the Academy of Medical Sciences (SGL023/1035 to S.Z.), the Medical Research Council Institutional Impact Acceleration Account at King's College London (MR/X502923/1), the Engineering and Physical Sciences Research Council (EP/X525571/1), and the King's‐China Scholarship Council PhD Scholarship Programme (202108410182 to A.D.). Additional support was provided by the Centre for Host–Microbiome Interactions, Faculty of Dentistry, Oral & Craniofacial Sciences, King's College London.

The SECRETO Oral study was funded by the Research Council of Finland (316777 and 355532 to S.P.; 340750 to P.P.), the Finnish Dental Society Apollonia, and the Sigrid Jusélius Foundation. The parent study was supported by the Research Council of Finland (286246, 318075, 322656 to J.P.), the Helsinki and Uusimaa Hospital District (TYH2014407, TYH2018318 to J.P.), the Sigrid Jusélius Foundation, and the Finnish Medical Foundation.

## Conflicts of Interest

The authors declare no conflicts of interest.

## Supporting information


**Figure S1:** Balanced sampling performed by random selection (*n* = 34 per group). Note: Participants from the SECRETO Oral study cohort were grouped according to periodontal and cerebrovascular disease status. Random sampling was performed within each disease‐status group to obtain balanced groups of 34 participants for the machine‐learning analysis.
**Figure S2:** Exploratory genus‐level correlations between subgingival microbial composition and subgingival LPS activity across disease‐status groups. Heatmap colours indicate Spearman correlation coefficients between genus‐level CLR‐transformed subgingival microbial abundances and log10‐transformed subgingival LPS activity. Representative genera were selected from the strongest FDR‐significant positive and negative associations observed across disease‐status groups. Asterisks indicate associations significant after Benjamini‐Hochberg FDR correction within each group. Full genus‐level correlation results are provided in Table S3.
**Table S1:** Feature‐set comparison of machine‐learning model performance.
**Table S2:** Decision curve analysis: raw net‐benefit values of the weighted voting model.
**Table S3:** Exploratory genus‐level correlations between subgingival microbial composition and subgingival LPS activity across disease‐status groups.

## Data Availability

The data that support the findings of this study are available from the corresponding author upon reasonable request.
