# Supplementary material for: Machine Learning Stratification of Periodontal and Cerebrovascular Disease Status Using Salivary and Subgingival Lipopolysaccharide Activity
Source: J Cell Mol Med. 2026 Aug 26;30(16):e71337. doi: 10.1111/jcmm.71337 (PMC13508137; doi:10.1111/jcmm.71337)
Supplement: Supplementary file 1 [file JCMM-30-e71337-s001.docx]

***Figure S1. Balanced sampling performed by random selection (n = 34 per group).*** Note: Participants from the SECRETO Oral study cohort were grouped according to periodontal and cerebrovascular disease status. Random sampling was performed within each disease-status group to obtain balanced groups of 34 participants for the machine-learning analysis.

***Supplementary Figure S2. Exploratory genus-level correlations between subgingival microbial composition and subgingival LPS activity across disease-status groups.*** Heatmap colours indicate Spearman correlation coefficients between genus-level CLR-transformed subgingival microbial abundances and log10-transformed subgingival LPS activity. Representative genera were selected from the strongest FDR-significant positive and negative associations observed across disease-status groups. Asterisks indicate associations significant after Benjamini-Hochberg FDR correction within each group. Full genus-level correlation results are provided in Supplementary Table S3.

**Supplementary Table S1. Feature-set comparison of machine-learning model performance.**

| **Feature set** | **Model** | **Accuracy** | **Macro-F1** | **Macro-AUROC** |
| --- | --- | --- | --- | --- |
| Demographic only | LogReg_EN | 0.522 | 0.521 | 0.764 |
|  | LinearSVC_Cal | 0.537 | 0.536 | 0.743 |
|  | RandomForest | 0.522 | 0.523 | 0.750 |
|  | XGBoost | 0.463 | 0.466 | 0.708 |
|  | WeightedVote | 0.522 | 0.523 | 0.752 |
| LPS-derived variables only | LogReg_EN | 0.478 | 0.452 | 0.722 |
|  | LinearSVC_Cal | 0.493 | 0.428 | 0.737 |
|  | RandomForest | 0.412 | 0.378 | 0.730 |
|  | XGBoost | 0.426 | 0.421 | 0.704 |
|  | WeightedVote | 0.471 | 0.435 | 0.748 |
| Demographic + LPS | LogReg_EN | 0.640 | 0.635 | 0.857 |
|  | LinearSVC_Cal | 0.618 | 0.600 | 0.830 |
|  | RandomForest | 0.588 | 0.584 | 0.854 |
|  | XGBoost | 0.581 | 0.582 | 0.830 |
|  | WeightedVote | 0.610 | 0.605 | 0.855 |

Note: Performance metrics were estimated using the same preprocessing, stratified five-fold cross-validation strategy, and model-evaluation pipeline for each feature set. The demographic/behavioural feature set included age, sex, BMI, smoking status, heavy alcohol use, diabetes history, and hypertension. The LPS-derived feature set included integrated oral LPS activity (LPS_log_mean) and subgingival-to-salivary LPS activity ratio (LPS_log_ratio). Macro-averaged metrics gave equal weight to each disease-status group. AUROC, area under the receiver operating characteristic curve; BMI, body mass index; LPS, lipopolysaccharide.

**Supplementary Table S2. Decision curve analysis: raw net-benefit values of the weighted voting model.**

| Threshold | Treat all | NonP/NoStroke | NonP/Stroke | P/NoStroke | P/Stroke |
| --- | --- | --- | --- | --- | --- |
| 0.05 | 0.211 | 0.224 | 0.212 | 0.212 | 0.219 |
| 0.10 | 0.167 | 0.217 | 0.206 | 0.164 | 0.206 |
| 0.15 | 0.118 | 0.200 | 0.193 | 0.145 | 0.201 |
| 0.20 | 0.062 | 0.186 | 0.165 | 0.097 | 0.162 |
| 0.25 | 0.000 | 0.159 | 0.145 | 0.071 | 0.135 |
| 0.30 | -0.071 | 0.160 | 0.146 | 0.050 | 0.125 |
| 0.35 | -0.154 | 0.162 | 0.137 | 0.038 | 0.094 |
| 0.40 | -0.250 | 0.147 | 0.118 | 0.054 | 0.076 |
| 0.45 | -0.364 | 0.136 | 0.117 | 0.047 | 0.072 |
| 0.50 | -0.500 | 0.103 | 0.088 | 0.044 | 0.051 |
| 0.55 | -0.667 | 0.109 | 0.060 | -0.007 | 0.025 |
| 0.60 | -0.875 | 0.129 | 0.055 | -0.011 | 0.015 |
| 0.65 | -1.143 | 0.111 | 0.054 | -0.014 | 0.003 |
| 0.70 | -1.500 | 0.103 | 0.025 | 0.000 | -0.005 |
| 0.75 | -2.000 | 0.066 | 0.015 | 0.000 | 0.015 |
| 0.80 | -2.750 | 0.022 | 0.007 | 0.000 | 0.007 |
| 0.85 | -4.000 | 0.000 | 0.000 | 0.000 | 0.000 |
| 0.90 | -6.500 | 0.000 | 0.000 | 0.000 | 0.000 |
| 0.95 | -14.000 | 0.000 | 0.000 | 0.000 | 0.000 |

Note: Net benefit of the weighted voting model under a one-vs-rest formulation for each disease group, alongside the treat-all reference strategy, across threshold probabilities. The treat-none strategy has a net benefit of 0 at all thresholds. NonP, no periodontitis; P, periodontitis.

**Supplementary Table S3. Exploratory genus-level correlations between subgingival microbial composition and subgingival LPS activity across disease-status groups.**

| **Disease-status group** | **Taxon** | **Spearman rho** | **FDR q value** |
| --- | --- | --- | --- |
| Periodontitis+ / Stroke+ | Pseudopropionibacterium | -0.653 | 0.007 |
| Periodontitis+ / Stroke+ | Actinomyces | -0.571 | 0.036 |
| Periodontitis+ / Stroke+ | Kingella | -0.565 | 0.036 |
| Periodontitis+ / Stroke+ | Lautropia | -0.540 | 0.050 |
| Periodontitis+ / Stroke- | Actinomyces | -0.608 | 0.037 |
| Periodontitis+ / Stroke- | Rothia | -0.587 | 0.037 |
| Periodontitis- / Stroke+ | Haemophilus | -0.617 | 0.030 |
| Periodontitis- / Stroke+ | Actinomyces | -0.601 | 0.030 |
| Periodontitis- / Stroke+ | Rothia | -0.573 | 0.030 |
| Periodontitis- / Stroke+ | Comamonas | -0.563 | 0.030 |
| Periodontitis- / Stroke+ | Leptothrix | -0.552 | 0.030 |
| Periodontitis- / Stroke+ | Variovorax | -0.552 | 0.030 |
| Periodontitis- / Stroke+ | Campylobacter | -0.548 | 0.030 |
| Periodontitis- / Stroke+ | Lachnospiraceae_(G-2) | -0.534 | 0.030 |
| Periodontitis- / Stroke+ | Mogibacterium | 0.526 | 0.030 |
| Periodontitis- / Stroke+ | Capnocytophaga | -0.523 | 0.030 |
| Periodontitis- / Stroke+ | Cutibacterium | -0.523 | 0.030 |
| Periodontitis- / Stroke+ | Lawsonella | -0.520 | 0.030 |
| Periodontitis- / Stroke+ | Bacteroidetes_(G-5) | 0.515 | 0.030 |
| Periodontitis- / Stroke+ | Lactococcus | -0.509 | 0.030 |
| Periodontitis- / Stroke+ | Sphingomonas | -0.498 | 0.030 |
| Periodontitis- / Stroke+ | Saccharibacteria_(TM7)_(G-8) | -0.495 | 0.030 |
| Periodontitis- / Stroke+ | Selenomonas | 0.484 | 0.030 |
| Periodontitis- / Stroke+ | Acinetobacter | -0.483 | 0.030 |
| Periodontitis- / Stroke+ | Agrobacterium | -0.483 | 0.030 |
| Periodontitis- / Stroke+ | Alloscardovia | -0.483 | 0.030 |
| Periodontitis- / Stroke+ | Anaerococcus | -0.483 | 0.030 |
| Periodontitis- / Stroke+ | Bacillus | -0.483 | 0.030 |
| Periodontitis- / Stroke+ | Bacteroides | -0.483 | 0.030 |
| Periodontitis- / Stroke+ | Bacteroidetes_(G-4) | -0.483 | 0.030 |
| Periodontitis- / Stroke+ | Bacteroidetes_(G-6) | -0.483 | 0.030 |
| Periodontitis- / Stroke+ | Bosea | -0.483 | 0.030 |
| Periodontitis- / Stroke+ | Defluvibacter | -0.483 | 0.030 |
| Periodontitis- / Stroke+ | Desulfomicrobium | -0.483 | 0.030 |
| Periodontitis- / Stroke+ | Enterobacter | -0.483 | 0.030 |
| Periodontitis- / Stroke+ | Enterococcus | -0.483 | 0.030 |
| Periodontitis- / Stroke+ | Escherichia | -0.483 | 0.030 |
| Periodontitis- / Stroke+ | Flavitalea | -0.483 | 0.030 |
| Periodontitis- / Stroke+ | Gracilibacteria_(GN02)_(G-2) | -0.483 | 0.030 |
| Periodontitis- / Stroke+ | Jonquetella | -0.483 | 0.030 |
| Periodontitis- / Stroke+ | Klebsiella | -0.483 | 0.030 |
| Periodontitis- / Stroke+ | Kocuria | -0.483 | 0.030 |
| Periodontitis- / Stroke+ | Lachnospiraceae_(G-10) | -0.483 | 0.030 |
| Periodontitis- / Stroke+ | Mobiluncus | -0.483 | 0.030 |
| Periodontitis- / Stroke+ | Moraxella | -0.483 | 0.030 |
| Periodontitis- / Stroke+ | Neisseriaceae_(G-1) | -0.483 | 0.030 |
| Periodontitis- / Stroke+ | Peptoniphilaceae_(G-1) | -0.483 | 0.030 |
| Periodontitis- / Stroke+ | Peptostreptococcaceae_(XI)(G-3) | -0.483 | 0.030 |
| Periodontitis- / Stroke+ | Peptostreptococcaceae_(XI)(G-8) | -0.483 | 0.030 |
| Periodontitis- / Stroke+ | Propionibacteriaceae_(G-2) | -0.483 | 0.030 |
| Periodontitis- / Stroke+ | Proteus | -0.483 | 0.030 |
| Periodontitis- / Stroke+ | Pseudomonas | -0.483 | 0.030 |
| Periodontitis- / Stroke+ | Ruminococcaceae_(G-3) | -0.483 | 0.030 |
| Periodontitis- / Stroke+ | Simonsiella | -0.483 | 0.030 |
| Periodontitis- / Stroke+ | Stenotrophomonas | -0.483 | 0.030 |
| Periodontitis- / Stroke+ | Syntrophomonadaceae_(VIII)(G-1) | -0.483 | 0.030 |
| Periodontitis- / Stroke+ | Saccharibacteria_(TM7)_(G-4) | -0.480 | 0.031 |
| Periodontitis- / Stroke+ | Butyrivibrio | 0.471 | 0.036 |
| Periodontitis- / Stroke+ | Mollicutes_(G-2) | -0.460 | 0.041 |
| Periodontitis- / Stroke+ | Peptoniphilaceae_(G-2) | -0.460 | 0.041 |
| Periodontitis- / Stroke+ | Sneathia | -0.460 | 0.041 |
| Periodontitis- / Stroke+ | Parascardovia | -0.450 | 0.047 |
| Periodontitis- / Stroke+ | Staphylococcus | -0.448 | 0.047 |
| Periodontitis- / Stroke+ | Veillonellaceae_(G-1) | 0.445 | 0.049 |
| Periodontitis- / Stroke- | Bacteroidetes_(G-7) | -0.622 | 0.010 |
| Periodontitis- / Stroke- | Peptoniphilaceae_(G-2) | -0.622 | 0.010 |
| Periodontitis- / Stroke- | Peptostreptococcaceae_(XI)(G-3) | -0.622 | 0.010 |
| Periodontitis- / Stroke- | Cutibacterium | -0.600 | 0.010 |
| Periodontitis- / Stroke- | Peptostreptococcaceae_(XI)(G-6) | -0.586 | 0.010 |
| Periodontitis- / Stroke- | Pseudomonas | -0.581 | 0.010 |
| Periodontitis- / Stroke- | Actinomyces | -0.570 | 0.010 |
| Periodontitis- / Stroke- | Agrobacterium | -0.569 | 0.010 |
| Periodontitis- / Stroke- | Bosea | -0.569 | 0.010 |
| Periodontitis- / Stroke- | Comamonas | -0.569 | 0.010 |
| Periodontitis- / Stroke- | Flavitalea | -0.569 | 0.010 |
| Periodontitis- / Stroke- | Simonsiella | -0.569 | 0.010 |
| Periodontitis- / Stroke- | Cryptobacterium | -0.562 | 0.010 |
| Periodontitis- / Stroke- | Acinetobacter | -0.560 | 0.010 |
| Periodontitis- / Stroke- | Veillonellaceae_(G-1) | 0.558 | 0.010 |
| Periodontitis- / Stroke- | Lachnospiraceae_(G-2) | -0.554 | 0.010 |
| Periodontitis- / Stroke- | Neisseriaceae_(G-1) | -0.553 | 0.010 |
| Periodontitis- / Stroke- | Selenomonas | 0.536 | 0.010 |
| Periodontitis- / Stroke- | Peptoniphilaceae_(G-1) | -0.531 | 0.010 |
| Periodontitis- / Stroke- | Alloscardovia | -0.524 | 0.010 |
| Periodontitis- / Stroke- | Anaerococcus | -0.524 | 0.010 |
| Periodontitis- / Stroke- | Bacillus | -0.524 | 0.010 |
| Periodontitis- / Stroke- | Bacteroidetes_(G-4) | -0.524 | 0.010 |
| Periodontitis- / Stroke- | Bacteroidetes_(G-6) | -0.524 | 0.010 |
| Periodontitis- / Stroke- | Defluvibacter | -0.524 | 0.010 |
| Periodontitis- / Stroke- | Desulfomicrobium | -0.524 | 0.010 |
| Periodontitis- / Stroke- | Enterobacter | -0.524 | 0.010 |
| Periodontitis- / Stroke- | Enterococcus | -0.524 | 0.010 |
| Periodontitis- / Stroke- | Escherichia | -0.524 | 0.010 |
| Periodontitis- / Stroke- | Gracilibacteria_(GN02)_(G-2) | -0.524 | 0.010 |
| Periodontitis- / Stroke- | Klebsiella | -0.524 | 0.010 |
| Periodontitis- / Stroke- | Kocuria | -0.524 | 0.010 |
| Periodontitis- / Stroke- | Lachnospiraceae_(G-10) | -0.524 | 0.010 |
| Periodontitis- / Stroke- | Lawsonella | -0.524 | 0.010 |
| Periodontitis- / Stroke- | Mobiluncus | -0.524 | 0.010 |
| Periodontitis- / Stroke- | Mollicutes_(G-1) | -0.524 | 0.010 |
| Periodontitis- / Stroke- | Mollicutes_(G-2) | -0.524 | 0.010 |
| Periodontitis- / Stroke- | Moraxella | -0.524 | 0.010 |
| Periodontitis- / Stroke- | Peptostreptococcaceae_(XI)(G-8) | -0.524 | 0.010 |
| Periodontitis- / Stroke- | Propionibacteriaceae_(G-1) | -0.524 | 0.010 |
| Periodontitis- / Stroke- | Propionibacteriaceae_(G-2) | -0.524 | 0.010 |
| Periodontitis- / Stroke- | Proteus | -0.524 | 0.010 |
| Periodontitis- / Stroke- | Pseudoramibacter | -0.524 | 0.010 |
| Periodontitis- / Stroke- | Ruminococcaceae_(G-3) | -0.524 | 0.010 |
| Periodontitis- / Stroke- | Saccharibacteria_(TM7)_(G-4) | -0.524 | 0.010 |
| Periodontitis- / Stroke- | Saccharibacteria_(TM7)_(G-8) | -0.524 | 0.010 |
| Periodontitis- / Stroke- | Sphingomonas | -0.524 | 0.010 |
| Periodontitis- / Stroke- | Staphylococcus | -0.524 | 0.010 |
| Periodontitis- / Stroke- | Syntrophomonadaceae_(VIII)(G-1) | -0.524 | 0.010 |
| Periodontitis- / Stroke- | Parascardovia | -0.521 | 0.011 |
| Periodontitis- / Stroke- | Sneathia | -0.510 | 0.013 |
| Periodontitis- / Stroke- | Desulfovibrio | -0.508 | 0.013 |
| Periodontitis- / Stroke- | Scardovia | -0.491 | 0.018 |
| Periodontitis- / Stroke- | Granulicatella | -0.483 | 0.021 |
| Periodontitis- / Stroke- | Bacteroides | -0.481 | 0.021 |
| Periodontitis- / Stroke- | Catonella | 0.472 | 0.024 |
| Periodontitis- / Stroke- | Saccharibacteria_(TM7)_(G-2) | -0.467 | 0.027 |
| Periodontitis- / Stroke- | Lactococcus | -0.462 | 0.028 |
| Periodontitis- / Stroke- | Rothia | -0.461 | 0.028 |
| Periodontitis- / Stroke- | Haemophilus | -0.452 | 0.033 |
| Periodontitis- / Stroke- | Stenotrophomonas | -0.444 | 0.036 |
| Periodontitis- / Stroke- | Pedobacter | -0.443 | 0.036 |
| Periodontitis- / Stroke- | Jonquetella | -0.430 | 0.045 |
| Periodontitis- / Stroke- | Acidipropionibacterium | -0.425 | 0.047 |

Note: Spearman correlations were calculated between genus-level CLR-transformed subgingival microbial abundances and log10-transformed subgingival LPS activity within each disease-status group in the balanced trimmed analytical sample. P values were adjusted using the Benjamini-Hochberg false discovery rate correction within each group. Full results are shown for all tested genera, with FDR-significant associations indicated.
